# Redescription of *Tripaphylus musteli* (van Beneden, 1851) (Copepoda: Sphyriidae) and the relegation of *Paeon* Wilson, 1919 to synonymy with *Tripaphylus* Richiardi in Anonymous, 1878

**DOI:** 10.1007/s11230-017-9734-4

**Published:** 2017-06-01

**Authors:** George W. Benz, Geoffrey A. Boxshall

**Affiliations:** 1 Evolution and Ecology Group, Biology Department, Middle Tennessee State University, Murfreesboro, TN 37132 USA; 2 Department of Life Sciences, Natural History Museum, Cromwell Road, London, SW7 5BD UK

## Abstract

*Tripaphylus musteli* (van Beneden, 1851) (Copepoda, Siphonostomatoida, Sphyriidae) is redescribed from an adult female collected from the branchial chamber of a starry smooth-hound, *Mustelus asterias* Cloquet (Carcharhiniformes, Triakidae), captured in the English Channel off Portland, UK. The new account of *T. musteli* is the first based on a complete adult female and highlighted the lack of a robust distinction separating *Tripaphylus* Richiardi, in Anonymous, 1878 and *Paeon* Wilson, 1919 prompting us to relegate *Paeon* to a junior subjective synonym of *Tripaphylus*. In the light of this synonymy the eight former species of *Paeon* are transferred to *Tripaphylus* as follows: *T. ferox* (Wilson, 1919) new combination, *T. elongatus* (Wilson, 1932) new combination, *T. vassierei* (Delamare Deboutteville & Nuñes-Ruivo, 1954) new combination, *T. lobatus* (Kirtisinghe, 1964) new combination, *T. asymboli* (Turner, Kyne & Bennett, 2003) new combination, *T. versicolor* (Wilson, 1919) new combination, *T. australis* (Kabata, 1993) new combination, and *T. triakis* (Castro Romero, 2001) new combination. Comparisons between terminology used in this report and that in the literature indicate that all transformed adult females of *Tripaphylus* probably possess a full complement of cephalic appendages and maxillipeds. All limbs, with the exception of the maxillae share a general morphological similarity to the corresponding appendages of conspecific males. The maxilla of the transformed adult female of *Tripaphylus* is a small digitiform protuberance associated with a swelling in some species.

## Introduction

Adult females of *Tripaphylus* Richiardi, in Anonymous, 1878 and *Paeon* Wilson, 1919 (Copepoda, Siphonostomatoida, Sphyriidae) exclusively infect the walls of the buccal and branchial chambers of elasmobranch fishes (Wilson, 1919, 1932). Fully developed adult females are highly transformed relative to their corresponding adult males. They are mesoparasites (*sensu* Kabata, 1979), living with the head and neck region embedded in the host while the trunk and ovisacs trail free in the surrounding water (Wilson, 1919). This lifestyle allows the female to attain large body size, while being firmly attached in a partially protected microhabitat that provides hatching larvae with access to the external environment for dispersal. In addition, the infection site also allows for blood feeding with access to the large blood vessels servicing the gills. In this paper we redescribe *Tripaphylus musteli* (van Beneden, 1851) from an adult female specimen collected from the branchial chamber of the type host, a starry smooth-hound, *Mustelus asterias* Cloquet. We also recognise *Paeon* as a junior subjective synonym of *Tripaphylus*, and provide comments aimed at standardizing the interpretation of the paired cephalosomic appendages across *Tripaphylus* species.

## Materials and methods

The starry-hound *Mustelus asterias* was captured by trawling and examined after spending several weeks alive in a holding tank at the Native Marine Species Centre, Portland, UK. The host was killed and the copepod was excavated from the host and fixed in 70% ethanol. The specimen was photographed before being cleared in lactic acid. After clearing, the specimen was observed on a Leitz Diaplan microscope using differential interference contrast. Drawings were made using a drawing tube; measurements were made using a stage micrometer. Anatomical terminology conforms mostly to Kabata (1979) and Huys & Boxshall (1991). Names of hosts follow FishBase (Froese & Pauly, 2017).

**Order Siphonostomatoida Burmeister, 1835**

**Family Sphyriidae Wilson,** 1919

***Tripaphylus musteli*** **(van Beneden,** **1851** **)**

Syns *Lerneonema musteli* van Beneden, 1851 of van Beneden (1851a, b) and Vogt (1877); *Trypaphylum musteli* (van Beneden, 1851) of Anonymous (1878), Wilson (1919), van Oorde-de-Lint & Schuurmans Stekhoven (1936), Kirtisinghe (1964) and Pillai (1985); *Tripaphylus musteli* (van Beneden, 1851) of Richiardi (1880), Valle (1880), Carus (1885), Brian (1906), Scott & Scott (1913), Yamaguti (1963), Kabata (1979), Kazachenko (2001), Boxshall & Halsey (2004); *Lernaeenicus musteli* (van Beneden, 1851) of Bassett-Smith (1899) and Scott (1904)

*Host and locality of new specimen*: Starry smooth-hound, *Mustelus asterias* Cloquet (Carcharhiniformes, Triakidae) captured in English Channel off Portland, UK; host kept alive in holding tank until examined on 17 January 2014.

**Fig. 1:**
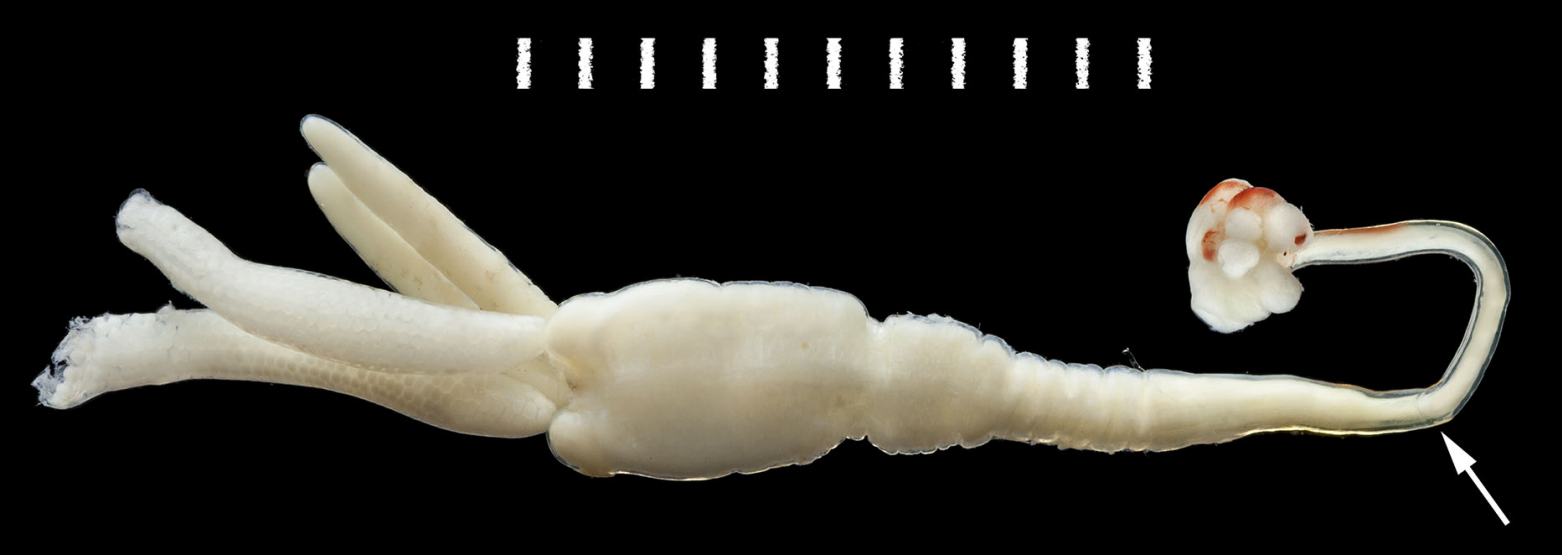
*Tripaphylus musteli* (van Beneden, 1851) (Siphonostomatoida, Sphyriiidae) transformed ovigerous female from a starry smooth-hound *Mustelus asterias* (Carcharhiniformes, Triakidae) captured in the English Channel off Portland, UK. *Scale-bar*: 1 cm divided into mm

*Material examined*: Ovigerous female (no attached male); neck of specimen broken during dissection but both parts recovered and restored digitally in Fig. 1; deposited in the Natural History Museum, London (Registration number NHMUK 2014.23).

*Other specimens examined*: Two headless ovigerous females (no attached male), from *M. mustelus* (*M. vulgaris* on label), Caernarvon Bay, UK, presented by T. Scott and A. Scott (BM(NH) Registration numbers 1913.9.18.324–325).

*Site in host*: Partially embedded in branchial chamber wall of host beyond distal perimeter of gill filament array.

### Description (Figs. 1–3)

**Fig. 2:**
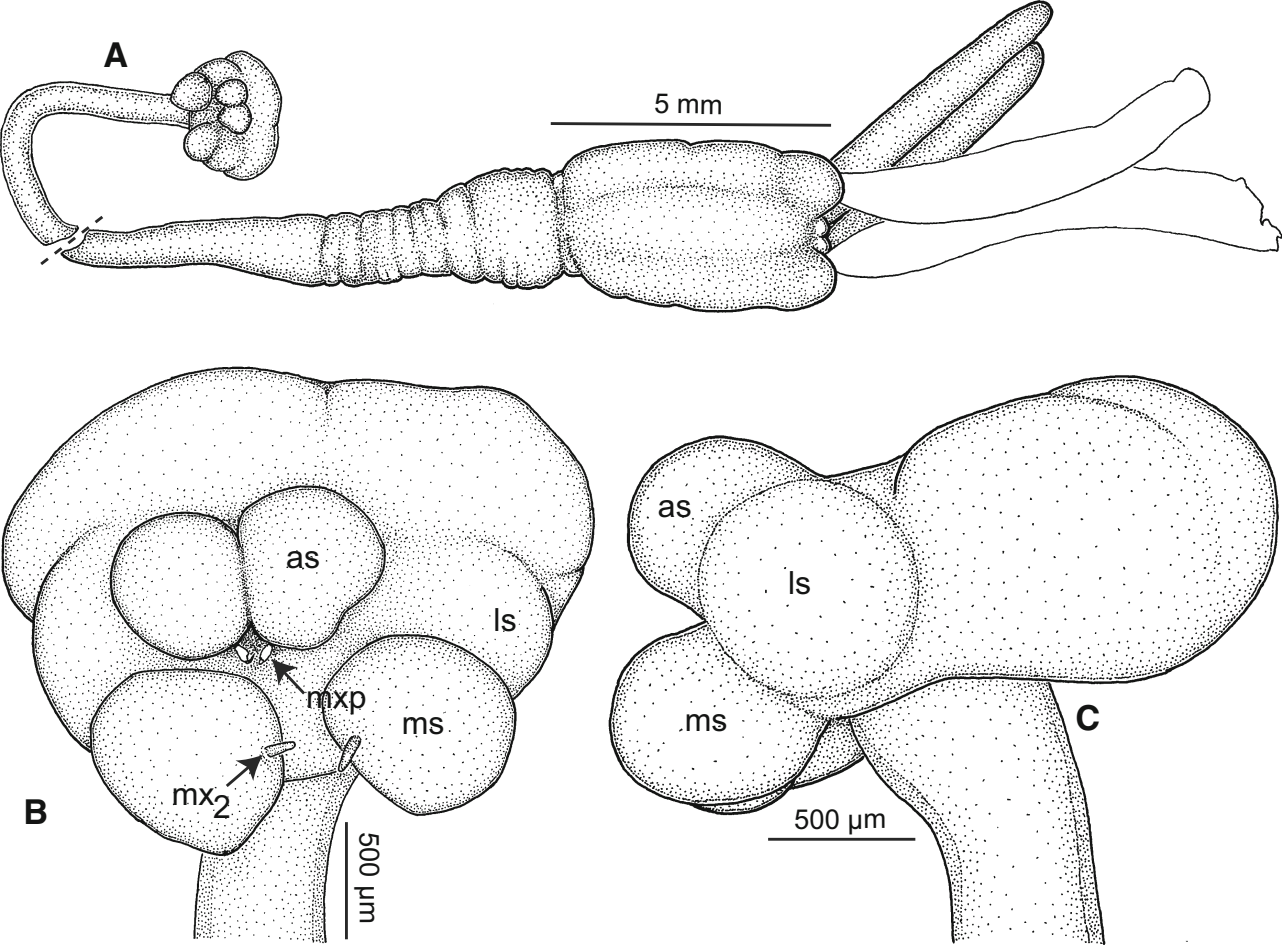
*Tripaphylus musteli* (van Beneden, 1851) A, General habitus; decapitated specimen with neck aligned (at dashed line) to approximate intact condition; head in ventral view, trunk in dorsal view; eggs within ovisacs not illustrated; B, Cephalothorax (head), ventral view; C, Left lateral view. *Abbreviations:* as, antennal swelling; ls, lateral swelling; ms, maxillary swelling; mx_2_, maxilla; mxp, maxilliped

*Transformed adult female*. Body (Figs. 1, 2A) comprising cephalothorax, neck, and trunk followed by small abdomen bearing pair of posterior processes; neck exhibiting torsion along longitudinal axis. Body dimensions (mm); reported as single measurements from complete specimen followed in some instances by measurements of headless Natural History Museum specimens BM(NH) 1913.918.324 and 1913.9.18.325: total length (cephalothorax tip to abdomen tip excluding posterior processes) 22.3, cephalothorax length 2.4, cephalothorax width 1.6, neck length 12.7, neck width 1.4, trunk length (neck boundary to abdomen base) 7.5 (6.7, 8.4), trunk width 3.2 (3.0, 3.3), abdomen length (not including posterior processes) 0.3 (0.3, 0.4), abdomen width 0.5 (0.4, 0.5), right posterior process length 5.6 (4.2, 6.6), right posterior process width 0.7 (0.7, 0.8), left posterior process length 6.1 (5.6, 6.5), left posterior process width 0.8 (0.7, 0.8), right ovisac (incomplete, tip ruptured) length 7.7, right ovisac width 1.2, left ovisac length 7.2, left ovisac width 1.3. Body regions of complete specimen expressed as percentage of total length: head length 10.8%, neck length 57.0%, trunk length 30.1%, abdomen length (not including posterior processes) 1.4%, right posterior process length 27.0%, left posterior process length 27.0%.

**Fig. 3:**
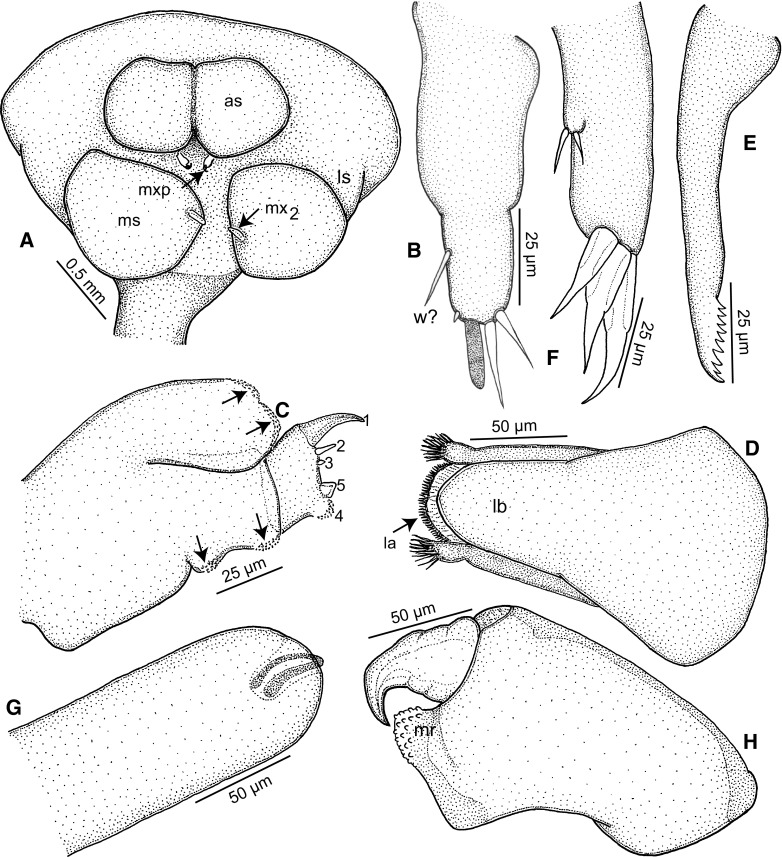
*Tripaphylus musteli* (van Beneden, 1851). A, Cephalothorax (head), slightly different ventral view than that depicted in Fig. 1B; B, Antennule; C, Antenna; *arrows* indicate denticulate patches; numbers correspond to element identification convention of Kabata (1979); D, Mouth tube, anterior view; E, Mandible; F, Maxillule, lateral view; G, Maxilla; H, Maxilliped. *Abbreviations:* as, antennal swelling; la, labium; lb, labrum; ls, lateral swelling; mr, myxal region of maxilliped; ms, maxillary swelling; mx_2_, maxilla; mxp, maxilliped; w?, whip?

Cephalothorax bulbous in ventral view (Figs. 2A–B, 3A), with 2 pairs of well-defined ventral swellings along midline, less-defined pair of lateral swellings, deeper than tall in lateral view (Fig. 2C), wider than tall in dorsal view with slight medial indentation along anterior border (Fig. 2B). First pair of well-defined swellings (antennary swellings) meeting at midline, obscuring antennules, antennae, mouth tube containing mandibles and maxillules (Figs. 2B, 3A). Second pair of well-defined, slightly larger swellings (maxillary swellings; Figs. 2B, 3A), positioned posterior to and lateral to antennary swellings, with gap between medial aspects; each swelling bearing small digitiform maxilla distally. Subtle lateral swellings (Figs. 2B, C, 3A) expressed, flanking antennary and maxillary swellings. Neck (Fig. 2A) cylindrical, anterior portion with smooth border, posterior portion with wrinkled cuticle (possibly due to contraction post-mortem); diameter expanding slightly at junction with head and to greater degree toward confluence with trunk. Trunk (Figs. 1, 2A) about twice as long as wide, slightly compressed dorsoventrally, width constant along most of length, posterior margin with small lateral lobes. Abdomen (Fig. 2A) represented by small tubercle bearing 2 cylindrical and blunt tipped posterior processes ventrally; posterior processes ventral to genital and oviduct openings. Ovisacs (Fig. 1A) cylindrical, longer than posterior processes, egg arrangement multiseriate, eggs spherical.

Coloration of live specimen off white, with red blotches primarily along portions of upper neck and ventral surface of head; recently fixed specimen (NHMUK 2014.23) off white with red blotches; older fixed specimens (BMNH 1913.9.18.324–325) pale brown with blackish blotches on neck, corresponding to red patches illustrated in Scott & Scott (1913: pl. LI, Figure 1).

Antennule (Fig. 3B) indistinctly 2-segmented with constriction delimiting basal part from narrower distal region; narrow region with long thin naked medial seta (whip?), apical region with small naked seta, aesthetasc and two long naked setae. Antenna (Fig. 3C) biramous, protopodal part unarmed; bulbous exopod one-segmented, shorter than endopod, apex ornamented with patches of denticles; endopod two-segmented, proximal segment with patches of denticles along medial border (arrowed in Fig. 3C), distal segment with robust hook 1, naked setae 2 and 5, small seta 3, and denticulated inflation 4 [using Kabata’s (1979) terminology].

Mouth tube (Fig. 3D) typical of sphyriids (Sphyriidae) and lernaeopodids (Lernaeopodidae), with anterior labrum and semi-circular (in cross-section) labium, latter with fringe of setules around mouth opening and swollen left and right anterolateral regions appearing as small projections tufted with setules in anterior view. Mandible (Fig. 3E) short, blade with nine teeth of about equal length. Maxillule (Fig. 3F) located lateral to mouth tube, bilobate; small outer lobe on lateral margin bearing 2 naked setae; inner lobe with 3 large naked apical setae, each with swollen base. Maxilla (Fig. 3G) small, digitiform, apically located on maxillary swelling (Fig. 3A); bearing blunt apical element nestled in cavity (element possibly retractile). Maxillipeds either side of midline between mouth tube and maxillary swellings (Fig. 3A); subchelate, each with broad corpus bearing denticulate myxal process opposing tip of subchela (Fig. 3H).

*Adult male.* None available for study; see Scott & Scott (1913: pp. 160–161, 229–230, pls. XLV (figure 6), XLIX (figures 1–7) for the most complete description (although inadequate by modern standards).

### Remarks

Van Beneden (1851a) established *Tripaphylus musteli* in *Lerneonema* Milne Edwards, 1840, as *Lerneonema musteli*. The essence of this account was republished during the same year in a different journal (van Beneden, 1851b). The species was mentioned again under its original name by Vogt (1877), but Vogt simply reviewed van Beneden’s account and re-used van Beneden’s original drawing of the male. The taxon was referred to again in an anonymous report (Anonymous, 1878) of the verbal proceedings of the Natural History Society of Toscania. Anonymous (1878) reported on an oral paper given by Richiardi, who referred to three complete specimens embedded in the hyoid region musculature of *Mustelus plebejus* Bonaparte (= *M. asterias* Cloquet, starry smooth-hound) and concluded that *Lernaeonema* [sic!] *musteli* van Beneden could not be placed in either *Lernaeenicus* Le Sueur or *Lernaeonema* [sic!] and instead belonged in a new genus related to *Sphyrion* Cuvier, 1830, for which Richiardi proposed the name *Trypaphylum*. Authorship was clearly attributed to Richiardi within the anonymous publication and thus Richiardi, in Anonymous, 1878 is the authority for *Trypaphylum*. Richiardi (1880) subsequently referred to the taxon in his systematic catalogue of parasitic crustaceans from aquatic animal hosts in Italy, but therein spelled it *Tripaphylus* and mentioned it as a new genus. Both spellings have subsequently been used for the genus; for example, *Trypaphylum* by Wilson (1919), van Oorde-de-Lint & Schuurmans Stekhoven (1936), Kirtisinghe (1964) and Pillai (1985), and *Tripaphylus* by Carus (1885), Scott & Scott (1913), Brian (1906), Yamaguti (1963), Kabata (1979), Dojiri & Deets (1988), Kazachenko (2001), Boxshall & Halsey (2004), Benz et al. (2006), and Gómez et al. (2010).

In his highly influential monograph, Kabata (1979) endorsed Richiardi’s (1880) spelling, *Tripaphylus*, in part because the earlier version was published anonymously, though validly under provisions of the International Code of Zoological Nomenclature (ICZN). Kabata construed the 1878 usage as a “lapsus calami (under ICZN Article 32(a)(ii))” (Kabata, 1979: p. 322). According to the fourth edition of the ICZN, to apply this ruling (now numbered 32.5.1) there must be in the original publication itself, “without recourse to any external source of information, clear evidence of an inadvertent error, such as a lapsus calami” (ICZN, 1999: p. 39). The evidence presented by Kabata (1979: p. 322) was, “because one other name mentioned in the same paragraph is spelt incorrectly”. Here we document greater use of *Tripaphylus* since 1880, and, in the interest of nomenclatural stability, we propose to follow Kabata (1979) and deem *Tripaphylus* to be a justified emendation under ICZN Article 33.2.3.1.

*Tripaphylus musteli* is a rarely collected and poorly known species. Van Beneden (1851a) based his original description on two incomplete females (decapitated with heads lost) and a male found attached to one of the females. Richiardi (in Anonymous, 1878) reported three intact females, but he gave no species description in that publication or in Richiardi (1880). Valle (1880) reported a single female from the branchial chamber of *Mustelus equestris* Bonaparte (= *Mustelus mustelus*) but provided no morphological details. Bassett-Smith (1899) mentioned specimens from the “gills” of *Mustelus vulgaris* (= *Mustelus mustelus*) in the collections of the British Museum but we have been unable to trace this material. Andrew Scott (1904) reported the species from the Irish Sea but merely noted its host and infection site. The description by Scott & Scott (1913) incorporated data from van Beneden’s (1851a, b) accounts in combination with new observations gained from examination of at least one headless female measuring 28 mm in length and of a male found attached to a female. It seems likely that Scott & Scott’s female description was based on the two headless specimens they presented to the Natural History Museum, London, however, no male was deposited by those authors. The headless female figured by Kabata (1979: figures 1459–1460) was one of the two specimens in the NHM collections. The present description of a complete specimen provides the first detailed description of the mouthparts and head of *T. musteli*, the type-species of the genus.

Kirtisinghe (1964) described a second species of *Tripaphylus* as *Trypaphylum hemigalei* based on adults of both sexes collected from the “gill arches” of *Hemigaleus balfouri* Day (= *Chaenogaleus macrostoma* (Bleeker), the hooktooth shark, Carcharhiniformes, Hemigaleidae) obtained from a Colombo market (Sri Lanka). He established the species based on the observation that the adult female of *T. hemigalei* was about half the length of *T. musteli* and possessed a head more transversely elliptical rather than round; furthermore, the males of each species differed regarding particular appendage characteristics. Kabata (1979) remarked that a robust decision regarding the validity of *T. hemigalei* as a species must await a detailed description of the head of *T. musteli*, and in doing so mentioned the conundrum that while the description of *T. musteli* by Scott & Scott (1913) was based on headless adult female specimens, those authors described the female in part as possessing a “Head rounded and furnished with cartilaginous horns” (Scott & Scott 1913: p. 160) and that description was adopted as part of their generic diagnosis for *Trypaphylus* (loc. cit.: p. 159). Kabata (1979) noted that while he was unaware of the origin of those details, they had been repeated by several subsequent authors, most notably Wilson (1919) and Yamaguti (1963). In fact, Kirtisinghe (1964) seemed to be aware of those details, as he referred to the head of *T. musteli* as being round. Furthermore, in addition to other general body features, the presence of an unbranched projection on each side of the head of the adult female of *T. hemigalei* likely was a key factor influencing Kirtisinghe’s designation of the generic affiliation for his new species. We too are unaware of the evidence underpinning Scott & Scott’s (1913) description of the head of *T. musteli*. Nevertheless, based on new details provided here, their remarks seem generally correct, in that at a certain magnification, the head of the adult female of *T. musteli* could be depicted as being rounded (Figs. 1, 2A) and in closer view the tiny digitiform maxillae carried on the maxillary swellings (the appendages least likely to be overlooked; Fig. 1B) could be interpreted as being horn-like. Unfortunately, the level of detail provided by Kirtisinghe (1964) regarding the appendages of *T. hemigalei* prevents meaningful comparisons with the details presented herein for *T. musteli*. Even so, we are confident that *T. hemigalei* is distinct from *T. musteli* based on differences in the general shape of the head; specifically, that of the adult female of *T. hemigalei* was noted by Kirtisinghe (1964) to be separated into a median and flanking lateral lobes with laterally projecting horns (maxillae) while our description reveals the head of the adult female of *T. musteli* as possessing well-defined pairs of antennary and maxillary swellings not observed on *T. hemigalei*.

## Discussion

In the phylogenetic analysis of the Sphyriidae by Benz et al. (2006), *Tripaphylus* formed an unresolved trichotomy with *Paeon* group A and a large lineage comprising *Norkus* Dojiri & Deets, 1988, *Paeon* group B, *Driocephalus* Raibaut, 1999, *Lophoura* Köllicker in Gegenbauer, Köllicker & Müller, 1853, *Sphyrion* Cuvier, 1830, *Periplexis* Wilson, 1919 and *Paeonocanthus* Kabata, 1965. In that analysis, group A species of *Paeon* (comprising the type-species *P. ferox* Wilson, 1919, plus *P. elongatus*, *P. vassierei* Delamare Deboutteville & Nuñes-Ruivo, 1954, *P. lobatus* Kirtisinghe, 1964 and *P. asymboli* Turner et al., 2003), and group B species of *Paeon* (comprised of *P. versicolor* Wilson, 1919, *P. australis* Kabata, 1993 and *P. triakis* Castro Romero, 2001), were distinguished from one another based on the form of the trunk and its boundary with the neck, defined as a single character. In *Paeon* group A species the trunk was noted as being “appreciably longer than wide” and “merges gradually with neck”, whereas in *Paeon* group B species the trunk was depicted as being “broad, roughly orbicular or rhomboid in dorsal view, well delimited from neck” (Benz et al., 2006: p. 3). In the most recent phylogenetic analysis of the family (Gómez et al., 2010), *Paeon* was treated as a single unit (not divided into group A and group B species), and *Tripaphylus* and *Paeon* were recovered as sister taxa. With regard to these two genera, the data matrix of Gómez et al. (2010: Table 2) was identical to that of Dojiri & Deets (1988: Appendix II). They were separated only by two characters relating to morphology of the cephalothorax, and the coding for *Trypaphylus* was presumably based on *T. hemigalei* using data extracted from Kirtisinghe (1964), given that the cephalothorax of the type-species *T. musteli* had not hitherto been documented.

All phylogenetic analyses of the sphyriids have been severely handicapped by the lack of detailed data on the structure of the cephalothoracic appendages of particular genera and molecular sequence data. Benz et al. (2006) commented that given the highly transformed morphology and seemingly reduced complexity of female sphyriids, it is likely that a robust phylogenetic hypothesis for the family will only be realized through molecular study.

Description herein of the mouthparts of the transformed females of *T. musteli* confirms their basic structural similarity to that of their corresponding males, as described in Scott & Scott (1913). Furthermore, we find no characters based on the mouthparts or any other feature that serve to discriminate at the genus level, between species of *Tripaphylus* and species of *Paeon*. The type-species of both genera share the same female body form, with a small head and long neck merging gradually into a trunk that bears a small abdomen carrying a pair of posteriorly projecting cylindrical processes originating ventrally. The head carries paired antennary and maxillary swellings in *T. musteli* and similar paired lobes are present in *P. ferox* Wilson, 1919, the type-species of *Paeon* (see Wilson, 1919: pl. 56, figure 55).

Given the absence of any character that can serve to discriminate between the type-species of *Tripaphylus* and *Paeon*, we conclude there is no justification in maintaining the validity of both genera. *Tripaphylus* has priority and we therefore propose that *Paeon* be recognized as its junior subjective synonym and that all *Paeon* species be transferred accordingly. The ten valid species of *Tripaphylus* resulting from this nomenclatorial change are: *T. musteli* (type-species) and *T. hemigalei*, plus *T. ferox* (Wilson, 1919) new combination, *T. elongatus* (Wilson, 1932) new combination, *T. vassierei* (Delamare Deboutteville & Nuñes-Ruivo, 1954) new combination, *T. lobatus* (Kirtisinghe, 1964) new combination, *T. asymboli* (Turner, Kyne & Bennett, 2003) new combination, *T. versicolor* (Wilson, 1919) new combination, *T. australis* (Kabata, 1993) new combination, and *T. triakis* (Castro Romero, 2001) new combination. The monophyletic status of *Tripaphylus*, as now constituted, needs to be tested to ascertain the status of the group B species of Benz et al. (2006).

Our understanding of *Tripaphylus* species has always been hampered by a lack of study specimens, damaged study specimens, and inadequate descriptive works that lack sufficient detail, especially regarding the appendages and other head features. Furthermore, the male has been reported for only four of the eight former members of *Paeon* (*T. elongatus* n. comb., *T. ferox* n. comb., *T. vaissierei* n. comb., and *T. versicolor* n. comb.) and the only detailed description available is that provided by Lewis (1966) for the male of *T. vaissierei* n. comb. Informed by that, as well as by details of the males of the first two members of *Tripaphylus*, see Scott & Scott (1913) and Kirtisinghe (1964), we know that males of *Tripaphylus* exhibit a general habitus similar in many respects to those of other sphyriids (see Dojiri & Deets, 1988; Moran & Piasecki, 1994) and especially lernaeopodids with a type-A male (*sensu* Kabata, 1979). However, the taxonomic significance of the appendages and other small cuticular structures of the *Tripaphylus* male is impossible to assess based on our current understanding. For example, while we noted minor antennule, antenna, and maxillule armature differences and maxilliped myxal region differences between males of *T. musteli*, *T. hemigalei* and *T. vaissierei* n. comb., based on the reports of, respectively, Scott & Scott (1913), Kirtisinghe (1964) and Lewis (1966), some of these differences could be the result of intraspecific variation or observation shortcomings only recognizable through further study and the examination of additional specimens.

Regarding the transformed adult female of *Tripaphylus* species (as now constituted), the literature includes only five original reports (Wilson, 1919, 1932; Kirtisinghe, 1964; Lewis, 1966; this report) that provide explicit details of at least some of the appendages; even so, the absence of detail lessens the comparative value of Wilson’s and Kirtisinghe’s contributions. Nevertheless, data suggest that the transformed adult females of all *Tripaphylus* species possess a full or near full complement of cephalosomic appendages, with all but the maxillae (see below) sharing general similarities with the corresponding appendages of conspecific males as well as with the corresponding appendages of post-metamorphosis adult female lernaeopodids; e.g. compare appendages reported herein and by Lewis (1966) with those of the many lernaeopodids reported by Kabata (1979). Here we re-interpret (Table 1) some of the morphological inferences in Scott & Scott (1913), Wilson (1919, 1932), Kirtisinghe (1964), Lewis (1966), and Turner et al. (2003).

**Table 1:** Comparison of terminology used for the appendages of *Tripaphylus* species in this and other published accounts (determined by examination of figured appendages and/or text descriptions)

Species	Appendage terminology used in present account						Reference
	Antennule	Antenna	Mandible	Maxillule	Maxilla	Maxilliped	
*T. ferox*	nr	Maxilla	nr	nr	Slender finger-like protuberance (text), migrated second antenna (figure legend)	Second maxilla	Wilson (1919)
*T. versicolor*	nr	nr	nr	Maxilla (text), first maxilla (figure legend)	Slender finger-like protuberance (text), migrated second antenna (figure legend)	Second maxilla	Wilson (1919)
*T. elongatus*	nr	Second antenna	nr	First mouth-part	nr	Second mouth part	Wilson (1932)
*T. vaissierei* ^a^	nr	nr	Mandible	Maxillule (?)	Maxilliped ?	Maxilla	Lewis (1966)
*T. asymboli*	nr	nr	nr	nr	Tentacular projection	nr	Turner et al. (2003)

^a^Lewis (1966) tentatively reported his *Tripaphylus* (as *Paeon*) specimens as *P. vaissierei*, but Kabata (1993) considered them to be conspecific with *P. lobatus*. We consider Lewis (1966) to have been correct in his determination

*Abbreviations: nr,* not reported; ? indicates reported uncertainty in original account

The swollen head processes of some *Tripaphylus* species obscure the antennules, antennae, mouth tube, maxillules and maxillae, and the confusion regarding the appendages of *Tripaphylus* post-metamorphosis females seems linked to the fact that, until this report, the maxillae have not been recognized as such, and have instead been reported as small digitiform protuberances or horns (Table 1). Dojiri & Deets (1988) proposed a similar structure (an indistinctly segmented digitiform protuberance bearing a subapical pore suspected of being a maxillary gland pore and an apical spine emanating from within a hollow) as the maxilla of *Norkus cladocephalus* Dojiri & Deets, 1988 (Sphyriidae) although that structure was not closely associated with a head swelling. The report of Moran & Piasecki (1994) is also relevant as it depicts the maxillae of *Sphyrion lumpi* (Krøyer, 1845) as fused swellings, each bearing an apical duct opening and small adjacent projection. Following our interpretation, the maxillae of the adult female of *Tripaphylus* migrate laterally from near the midline during metamorphosis, sometimes (e.g. *T. musteli*, *T. versicolor* n. comb. and *T. vaissierei* n. comb.) they are positioned posterolateral to the maxillipeds. That interpretation is founded in part on the assumption that young females of *Tripaphylus* likely share a similar general habitus with conspecific adult males, as has been shown for lernaeopodids (Kabata & Cousens 1973; Piasecki & Kuźmińska, 2007). Based on these findings, we hypothesise that transformed adult females of *Tripaphylus* species all possess a full complement of cephalic appendages and we consider it likely that some to many of those appendages remain to be discovered in congeners other than *T. musteli* and *T. hemigalei*.

Finally, in light of our better understanding of the morphology of *Tripaphylus* species and the decision to relegate *Paeon* to a junior subjective synonym of *Tripaphylus*, we offer an amended diagnosis for *Tripaphylus* as follows:

**Genus** ***Tripaphylus*** **Richiardi in Anonymous,** 1878

*Diagnosis*

Transformed (post-metamorphosis) adult female comprising bulbous head, paired (left and right) swellings on ventral surface of head, long and slender cylindrical neck, wider and somewhat dorsoventrally compressed trunk, with small abdominal tubercle bearing ventrally two long, simple, posteriorly projecting, cylindrical processes; neck sometimes exhibiting torsion. Ovisacs positioned dorsal to posterior processes, straight, multiseriate, containing spherical eggs. Antennules, antennae, mandibles within mouth tube, maxillules and maxillipeds typical of most sphyriids and lernaeopodids. Maxillae small, digitiform, located posterior to maxillipeds. Transformed adult female mesoparasitic, with head and much of neck embedded in host and remainder of body trailing free. Adult male typically found attached to abdominal region of transformed adult female. Adult male general habitus comprising cephalothorax and thoraco-genito-abdominal trunk. Cephalothorax about half body length and wider than trunk, caudal rami at apex of trunk. Antennules, antennae, mandibles, maxillules as in corresponding adult female. Maxillae subchelate, arising from common base. Maxillipeds chelate, arising from large common base such that tips often lie below tip of abdomen. Medial process projecting from ventral surface just anterior to maxillipeds.
